# Analytic time-of-flight positron emission tomography reconstruction: two-dimensional case

**DOI:** 10.1186/s42492-019-0035-4

**Published:** 2019-12-09

**Authors:** Gengsheng L. Zeng, Ya Li, Qiu Huang

**Affiliations:** 1 Department of Engineering, Utah Valley University, Orem, UT 84058 USA; 2 Department of Radiology and Imaging Sciences, University of Utah, Salt Lake City, UT 84108 USA; 3 Department of Mathematics, Utah Valley University, Orem, UT 84058 USA; 4 School of Biomedical Engineering, Shanghai Jiaotong University, Shanghai, 200240 China

**Keywords:** Positron emission tomography, Time-of-flight, Analytic reconstruction

## Abstract

In a positron emission tomography (PET) scanner, the time-of-flight (TOF) information gives us rough event position along the line-of-response (LOR). Using the TOF information for PET image reconstruction is able to reduce image noise. The state-of-the-art TOF PET image reconstruction uses iterative algorithms. Analytical image reconstruction algorithm exits for TOF PET which emulates the iterative Landweber algorithm. This paper introduces such an algorithm, focusing on two-dimensional (2D) reconstruction. The proposed algorithm is in the form of backprojection filtering, in which the backprojection is performed first, and then a 2D filter is applied to the backprojected image. For the list-mode data, the backprojection is carried out in the event-by-event fashion, and a profile function may be used along the projection LOR. The 2D filter depends on the TOF timing resolution as well as the backprojection profile function. In order to emulate the iterative algorithm effects, a Fourier-domain window function is suggested. This window function has a parameter, *k*, which corresponds to the iteration number in an iterative algorithm.

## Introduction

One of the advantages of using time-of-flight (TOF) technology is its ability to reduce the image noise [1, 2]. The state-of-the-art TOF positron emission tomography (PET) image reconstruction methodology is to use the iterative algorithms such as TOF ordered-subset expectation-maximization algorithms [2].

The filtered backprojection (FBP) algorithm is not a preferred method nowadays, due to the concerns of potential larger noise amplification with the FBP algorithm than the iterative algorithms. These concerns are not well-founded. As we demonstrated before, the FBP algorithm should perform as well as an iterative algorithm when the iteration number is emulated and the projection noise is modeled in the FBP algorithm [3, 4]. We believe that analytical image reconstruction algorithm can achieve the same noise level as a linear iterative image reconstruction algorithm, e.g., the iterative Landweber algorithm. The same can be said to the backprojection filtering (BPF) algorithm, which is an analytic algorithm that performs backprojection first and then performs filtering [5]. For the list-mode data, it is computationally more efficient to use a BPF algorithm than an FBP algorithm. We recommend use of a BPF algorithm so that it is fast, robust and rebinning error free. In the conventional BPF algorithm, the backprojected image does not have a finite support, and this makes the final filtering step not exact. However, for a TOF backprojector, the backprojected image has a finite support if the backprojection profile function has a finite support. The TOF BPF algorithm has a potential to have better accuracy if the TOF information is used. We will show in the later part of this paper that the TOF modified “ramp filter” is “more local” than the conventional ramp filter. Here, “more local” means that the spatial-domain convolution kernel's rolls-off rate is faster. In the BPF algorithm the ramp filter is often referred to as the ρ-filter; we will use the term “ramp filter” in this paper.

Fully three-dimensional (3D) TOF iterative reconstruction is computationally expensive. When the object is completely measured, rebinning methods are available to convert the 3D measurements into two-dimensional (2D) measurements, so that faster 2D image reconstruction can be performed [6, 7]. This paper will only focus on 2D image reconstruction.

The objective of this work is to develop an analytical image reconstruction algorithm for the TOF PET. This current paper develops a “first backproject, and then filter” (BPF) algorithm for the 2D TOF PET. This algorithm is computationally efficient as the conventional FBP algorithm and is able to regulate noise as the iterative reconstruction algorithm.

## Methods

The TOF timing resolution is not perfect. A time uncertainty of 100 p-seconds (ps) can be translated into a positional uncertainty of 1.5 cm full-width-at-half-maximum. This note will investigate the TOF BPF algorithm, only focusing on the 2D case. Some attempts of using analytic BFP and FBP algorithms for TOF PET have been reported, for example, in refs. [1, 2], respectively.

In TOP FBP algorithm, one can bin list-mode data in to view-by-view sinogram format in multiple sets; each set corresponding to a range of TOF time difference. One can also implement an event-by-event FBP algorithm, in which each event is replaced by the convolution kernel and then each of such kernel is backprojected into the image space with a profile function.

### List-mode TOF 2D backprojection point spread function

**Fig. 1:**
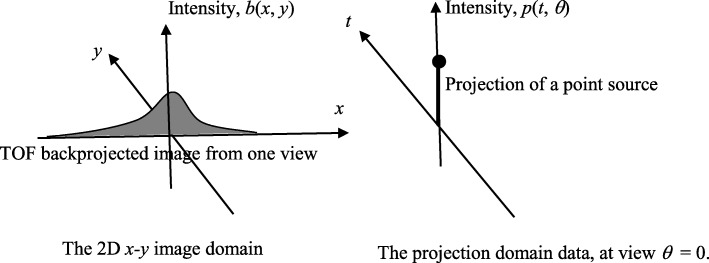
Projection of a point source at the origin (shown on the right) and the associated time-of-flight (TOF) backprojection (shown on the left). The TOF backprojection of a single event uses a profile function along the line-of-response

For list-mode data, a BPF algorithm is computationally less expensive than an FBP algorithm, because each event needs to be filtered and backprojected in FBP. In a list-mode TOF PET data BPF algorithm, we first backproject each measured event along the line-of-response (LOR) with a profile function, which can be a normalized Gaussian function centered at the estimated location by the TOF information. The standard deviation of the Gaussian function is related to the time uncertainty of the system. Figure 1 illustrates the TOF backprojection of the projection obtained from a point source at the center of the image plane. Then a 2D post filter is applied. This post filter will be derived by considering the point spread function (psf) next.

The traditional central slice theorem can be stated in the following expression in the Fourier domain:

1 $$ P\left(\omega, \theta \right)={F}_{polar}\left(\omega, \theta \right) $$

where *ω* is the frequency, *θ* is the detection view angle, *F*_*polar*_ is the 2D Fourier transform (in the polar coordinates) of the object *f*(*ρ*, *θ*), and *P*(*ω*, *θ*) is the one-dimensional (1D) Fourier transform of the parallel projections *p*(*t*, *θ*) of the object at view *θ*. In other words, *F*_*polar*_(*ω*, *θ*) = *F*_1D_ {*f*(*ρ*, *θ*)} and *P*(*ω*, *θ*) = *F*_1D_ {*p*(*t*, *θ*)}. A “slice” is a function defined on a straight line in the 2D plane.

The object *f*(*x, y*) can be considered as a ramp-filtered version of a backprojection image whose 2D Fourier domain representation (in the polar coordinates) is *B*_*polar*_:

2 $$ {F}_{polar}\left(\omega, \theta \right)=\mid \omega \mid \times {B}_{polar}\left(\omega, \theta \right) $$

where *B*_*polar*_(*ω*, *θ*) is the 2D Fourier transform of the pure backprojection (without ramp filtering) *b*_*polar*_(*ρ, θ*). In other words, *B*_*polar*_(*ω*, *θ*) = *F*_1D_ {*b*(*ρ*, *θ*)}. Combining Eqs. (1) and (2) yields

3 $$ P\left(\omega, \theta \right)=\mid \omega \mid \times {B}_{polar}\left(\omega, \theta \right) $$

that is,

4 $$ \frac{P\left(\omega, \theta \right)}{\mid \omega \mid }={B}_{polar}\left(\omega, \theta \right) $$

If the object is angular symmetric, Eq. (4) becomes

5 $$ \frac{P\left(\omega \right)}{\mid \omega \mid }={B}_{polar}\left(\omega \right) $$

Backprojection from one angle is equivalent to adding a “slice” *P*(*ω*) in the Fourier domain to *B*_*polar*_(*ω*, *θ*). The total effect of backprojection from all angles is

6 $$ \frac{P\left(\omega \right)}{\mid \omega \mid } $$

Next, we consider a point source at the origin, its projection and TOF backprojection. The projection and TOF backprojection are shown in Fig. 1. The TOF backprojection uses the profile function.

**Fig. 2:**
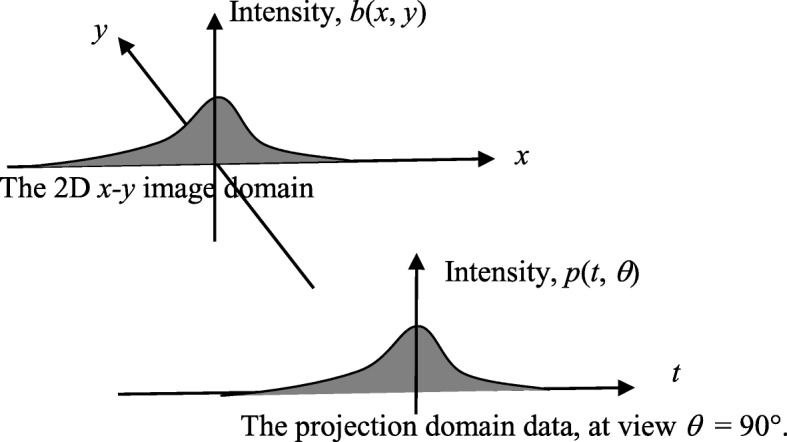
The time-of-flight (TOF) backprojector is equivalent to a backprojector, in which the projection (here, the TOF profile function) is first rotated by 90° and then is backprojected only to the line passing through the origin and parallel to the detector

To obtain the same effect, we can rotate the detector by 90° and convolve the projections, which is an impulse in our case, with a special convolution kernel. This convolution kernel is nothing but the TOF backprojection profile function. This TOF backprojector only backprojects to the line passing through the origin and parallel to the detector (Fig. 2).

**Fig. 3:**
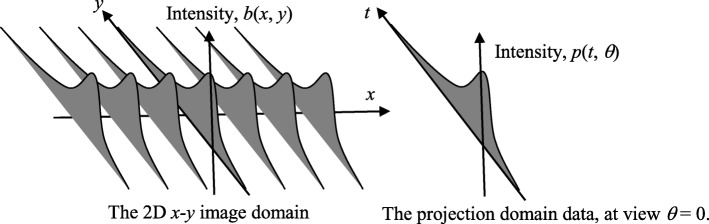
The conventional two-dimensional backprojection for the parallel-beam geometry. The conventional backprojection copies the function along the projection direction throughout the entire image space

We must point out that this TOF backprojector is different from the standard backprojector. The standard backprojector backprojects a function to the entire image plane (Fig. 3). The main difference between these two backprojectors is that the TOF backprojector only adds a “slice” to the image plane in the spatial domain.

The standard backprojector adds a “slice” in the Fourier domain; the TOF backprojector adds a “slice” in the spatial domain.

It is known that when we add a “slice” *P*(*ω*) at each view angle in the Fourier domain, the total effect for all angles is *P*(*ω*)/ ∣ *ω*∣. The duality of the previous sentence is as follows. When we add a “slice” *g*(*r*) at each view angle in the spatial domain, the total effect for all angles is *g*(*r*)/ ∣ *r*∣. Here, *g*(*r*) is a “slice” which represents the TOF backprojection profile function. The “total effect” is the psf that we are looking for. Thus, we have

7 $$ g(r)=r\times psf(r) $$

with *r* ≥ 0 in the polar coordinate system.

The tomography filter depends on the 2D psf in the TOF backprojected image. Using the Cartesion coordinate systems, let the 2D psf be *psf*(*x, y*), the true image be *f*(*x, y*), and the TOF backprojected image be *b*(*x, y*). Then their relationship is

8 $$ b\left(x,y\right)=f\left(x,y\right)\ast \ast psf\left(x,y\right) $$

where “ ∗∗ ” denotes the 2D convolution. If we select the normalized Gaussian function

9 $$ g(r)=\frac{1}{2\pi {\sigma}^2}{e}^{-\frac{r^2}{2{\sigma}^2}} $$

as the TOF backprojection profile function, with *r* being the variable along the LOR, the *psf*(*x, y*) can be evaluated as backprojection of Eq. (9) over 2π, which is the same as Eq. (10) by using Eq. (7):

10 $$ psf(r)=\frac{1}{2\pi {\sigma}^2r}{e}^{-\frac{r^2}{2{\sigma}^2}} $$

### 2D TOF BPF algorithm

Let the true 2D object be *f*_*polar*_(*r*, *θ*) in the polar coordinates and the TOF backprojected image be *b*_*polar*_(*r*, *θ*) in the polar coordinates. We have the relationship

11 $$ {b}_{polar}\left(r,\theta \right)={f}_{polar}\left(r,\theta \right)\ast \ast psf(r) $$

which is an equivalent form of Eq. (8) when the psf is only a function of *r*. A BPF algorithm is to deconvolve *b*_*polar*_(*r*, *θ*) with a 2D convolution filter *h*(*r*), which satisfies

12 $$ h(r)\ast \ast psf(r)=\delta (r) $$

with *δ*(*r*) being the Dirac delta function.

Evaluating the function *h*(*r*) can be achieved by using the 2D Fourier transform. Since the psf is not a function of angle *θ*, the 2D Fourier transform of the psf can be evaluated by the 1D Hankel transform as follows

13 $$ {\displaystyle \begin{array}{l}\underset{0}{\overset{\infty }{\int }}\underset{0}{\overset{2\pi }{\int }}\frac{\frac{1}{2\pi {\sigma}^2}{e}^{-\frac{r^2}{2{\sigma}^2}}}{r}{e}^{-2\pi i\left(\begin{array}{c}r\cos \varphi \\ {}r\sin \varphi \end{array}\right)\cdot \left(\begin{array}{c}\omega \cos {\varphi}_{\omega}\\ {}\omega \sin {\varphi}_{\omega}\end{array}\right)} rdr d\varphi \\ {}=\underset{0}{\overset{\infty }{\int }}\frac{\frac{1}{2\pi {\sigma}^2}{e}^{-\frac{r^2}{2{\sigma}^2}}}{r} rdr\underset{0}{\overset{2\pi }{\int }}{e}^{-2\pi i\cos \left(\varphi -{\varphi}_{\omega}\right)} d\varphi \\ {}=\underset{0}{\overset{\infty }{\int }}\frac{\frac{1}{2\pi {\sigma}^2}{e}^{-\frac{r^2}{2{\sigma}^2}}}{r} rdr\underset{0}{\overset{2\pi }{\int }}{e}^{-2\pi i\cos \varphi } d\varphi \\ {}=\underset{0}{\overset{\infty }{\int }}\frac{\frac{1}{2\pi {\sigma}^2}{e}^{-\frac{r^2}{2{\sigma}^2}}}{r}{J}_0\left(2\pi \omega r\right) rdr\\ {}=\frac{1}{2\pi {\sigma}^2}\underset{0}{\overset{\infty }{\int }}{e}^{-\frac{r^2}{2{\sigma}^2}}{J}_0\left(2\pi \omega r\right) dr\\ {}=\frac{1}{2\sqrt{2\pi }}{e}^{-{\left(\pi \sigma \omega \right)}^2}\times {I}_0\left({\left(\pi \sigma \omega \right)}^2\right),\end{array}} $$

where *J*_0_ is the Bessel function of the first kind with order 0 defined as ${J}_0(z)=\frac{1}{2\pi }{\int}_0^{2\pi }{e}^{- iz\cos \varphi } d\varphi$ and *I*_0_ is the modified Bessel function of the first kind with order 0 defined as ${I}_0(z)=\frac{1}{\pi }{\int}_0^{\pi }{e}^{-z\cos \varphi } d\varphi$. The first line in Eq. (13) is the 2D Fourier transform using the polar coordinates. The fourth line in Eq. (13) is the 1D Hankel transform. The last line in Eq. (13) is from an integration formula 6.618.1 in ref. [8]: $\underset{0}{\overset{\infty }{\int }}{e}^{-\alpha {x}^2}{J}_0\left(\beta x\right) dx=\frac{\sqrt{\pi }}{2\sqrt{\alpha }}{e}^{-\frac{\beta^2}{8\alpha }}{I}_0\left(\frac{\beta^2}{8\alpha}\right)$. The Eq. (13) is called the transfer function for the projection and TOF backprojection procedure.

According to Eqs. (11) and (12), the post tomographic filter *H*(*ω*) expressed in the 2D Fourier domain (in the polar coordinates) can be obtained as reciprocal of the transfer function (13), normalized by forcing *H*(0) = 1, as

14 $$ H\left(\omega \right)=\frac{e^{{\left(\pi \sigma \omega \right)}^2}}{I_0\left({\left(\pi \sigma \omega \right)}^2\right)}. $$

In a list-mode TOF BPF algorithm, the list-mode events are first backprojected into the image space using a profile function. Then a 2D filter (14) is applied to the backprojected image. Eq. (14) is in the Fourier domain and contains a special function *I*_0_. A close approximation of Eq. (14) using elementary functions is

15 $$ H\left(\omega \right)=\frac{e^{{\left(\pi \sigma \omega \right)}^2}}{I_0\left({\left(\pi \sigma \omega \right)}^2\right)}\approx \sqrt{1+{\left(2\pi \sigma \omega \right)}^2}. $$

**Fig. 4:**
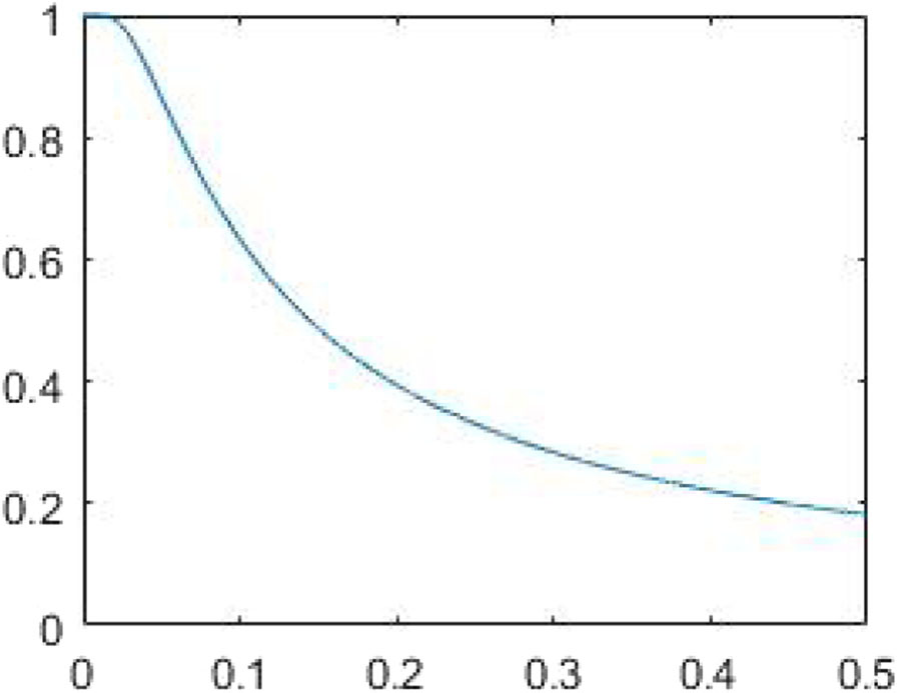
A typical plot for the noise-control Fourier-domain window function *W*(*ω*) with *k* = 1000 and *α* = 0.0001. The horizontal axis is the frequency *ω*: 0 ≤ *ω* ≤ 0.5. The vertical axis is the gain *W*(*ω*), which suggests a low-pass filter

Some examples of Eqs. (14) and (15) are shown in Fig. 4 in the first and second columns, respectively.

When *σ* is very large (i.e., the timing resolution is poor), the right-hand-side of Eq. (15) approaches to the ramp filter 2*πσ*|*ω*|, which is the case for non-TOF tomography. When *σ* is very small (i.e., the timing resolution is good), the right-hand-side of Eq. (15) approaches to the constant 1, which means that no filtering is needed and the image can be reconstructed by pure TOF backprojection.

**Fig. 5:**
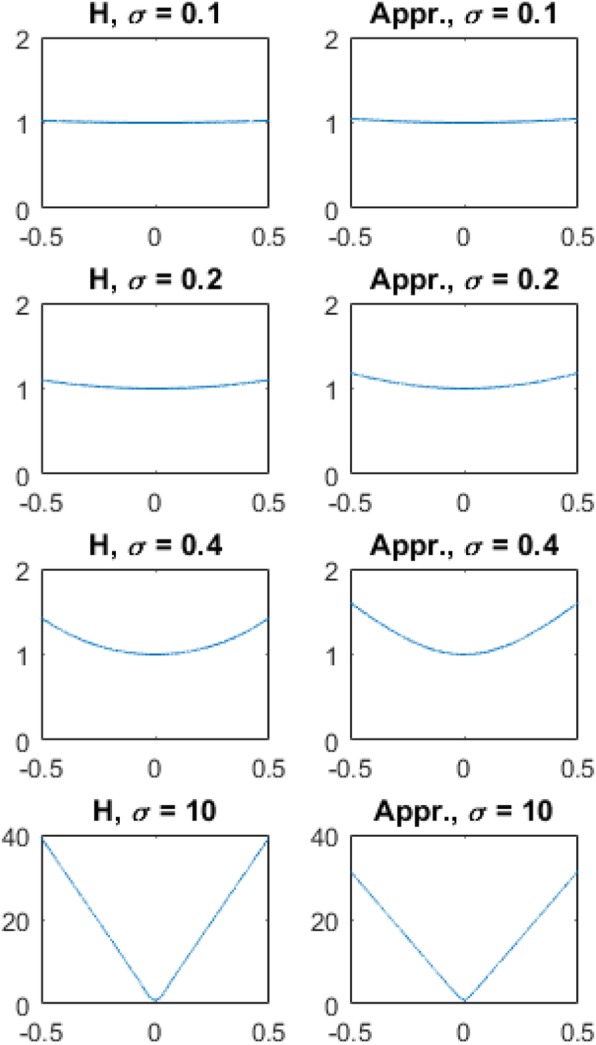
Some examples of the tomographic filters for the time-of-flight backprojection filtering algorithm with different σ values. Left: Fourier domain transfer functions (Eq. 14). Right: Square-root function approximations (Eq. 15)

Even though 2D Fourier-domain filtering can be implemented as 2D spatial-domain convolution, we are unable to find the closed-form convolution kernels directly from Eqs. (14) or (15). The convolution kernels can be evaluated numerically. Since the convolution kernel is radially symmetric, it is only necessary to evaluate the values of the kernel along a radial ray via the 1D Hankel transform, and the results are shown in Fig. 5.

### Noise-control window function in the BPF algorithm

Noise control for an analytic algorithm is achieved by application of a low-pass filter. Some low-pass filters work better than others. We developed a Landweber window function [4] that mimics the iterative Landweber algorithm and is suitable to for image reconstruction denoising. This window function has a parameter *k* that emulates the iteration number in the iterative Landweber algorithm. The window function is expressed as.

16 $$ W\left(\omega \right)=\Big\{{\displaystyle \begin{array}{cc}1&, \kern1em \omega =0\\ {}1-{\left(1-\frac{\alpha }{\omega}\right)}^k&, \kern1em \omega \ne 0\end{array}} $$

where the parameter *α* is chosen such that we always have ∣1 − *α*/*ω* ∣  < 1 for discrete samples of *ω*. A typical plot for *W*(*ω*) is shown below in Fig. 4. In algorithm implementation, the filter *H*(*ω*) in Eqs. (14) or (15) is realized as the product *H*(*ω*)*W*(*ω*).

## Results

The paper has derived a 2D TOF BPF image reconstruction algorithm. In this algorithm, the list-mode data is first backprojected according to a profile function, which can be assumed to be a normalized Gaussian function *g*(*r*) (9) in this computer simulation. Then the backprojected image is transformed into the 2D Fourier domain and is filtered by the post filter *H*(*ω*) presented in Eqs. (14) or (15). Figure 5 shows the comparison between Eqs. (14) and (15) for four different timing resolutions *σ*. When *σ* = 10, the filter *H* is almost a ramp filter. When *σ* = 0.1, the filter *H* is almost a constant.

**Fig. 6:**
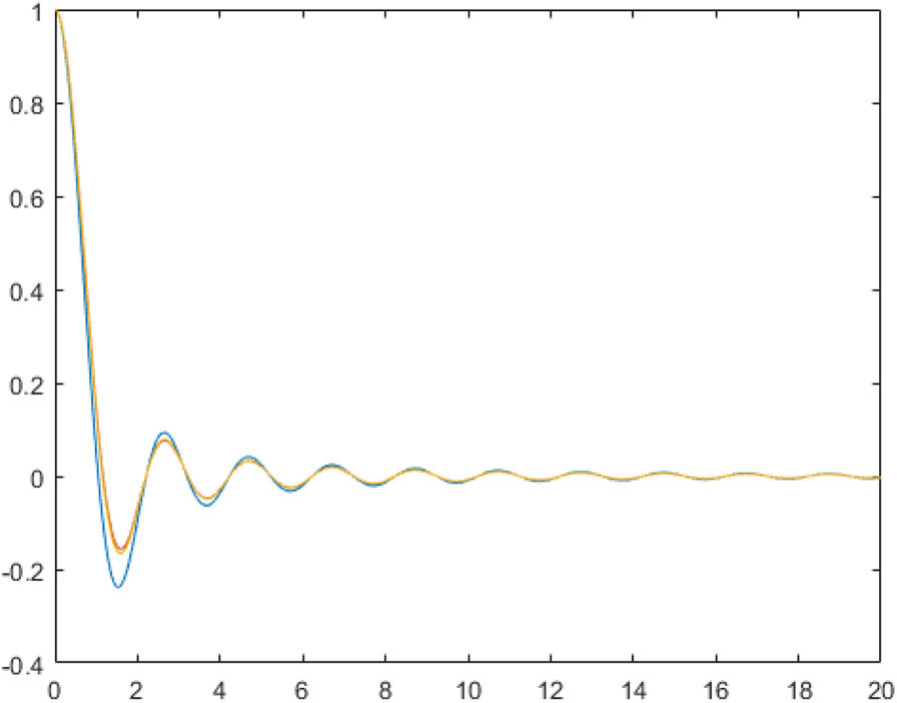
The two-dimensional convolution kernel is only a function of radial distance *r*. The blue curve is for the conventional ramp filter. The yellow and orange curves (on the top of each other) are for the *σ* = 0.5: one is computed with the true *H* and the other is computed square-root function approximation

We are unable to find a closed-form for spatial-domain convolution kernel *h*(*r*) that correspond to the transfer function *H*(*ω*) in Eqs. (14) or (15). When the 2D function *H* is angular symmetric, so is *h*. Therefore, the functions *h*(*r*) and *H*(*ω*) are related by the 1D Hankel transform pair. A numerical evaluation of the 1D Hankel transform can readily produce *h*(*r*) from a given *H*(*ω*). A numerically obtained results of *h*(*r*) from Eqs. (14) and (15) are shown in Fig. 5 for *σ* = 0.5. The conventional ramp filter’s convolution kernel is also displayed in Fig. 6 for the comparison purpose. It is observed that the conventional ramp filter has larger side-lobes than the other curves. As σ → 0, the convolution kernel tends to an impulse with no side-lobes.

Finally, a computer phantom study is presented to test the proposed analytical image reconstruction algorithm as follows. A computer simulated 2D Shepp-Logan head phantom was used to generate the parallel projections [9]. The phantom image was presented in a 128 × 128 array. The Radon projections had 180 views over 180°. Two sets of the projections were used for image reconstruction. One set was noiseless, and the other set was incorporated with Poisson noise.

The TOF timing resolution was modelled by a normalized Gaussian function with σ = 10. To simulate the TOF effect, the 1D projections were filtered with a filter *I*_0_(2*π*^2^*σ*^2^*ω*^2^)*epx*(−2*π*^2^*σ*^2^*ω*^2^). A conventional FBP algorithm was used to reconstruct image from the filtered projections. According to the central slice theorem, regular backprojection of the 1D filtered projection with the filter *I*_0_(2*π*^2^*σ*^2^*ω*^2^)*epx*(−2*π*^2^*σ*^2^*ω*^2^) gave the equivalent effect of the TOF backprojection with a Gaussian profile function. The above procedure gave the equivalent effect as obtaining the pure TOF backprojected image that has a psf given by Eq. (9).

**Fig. 7:**
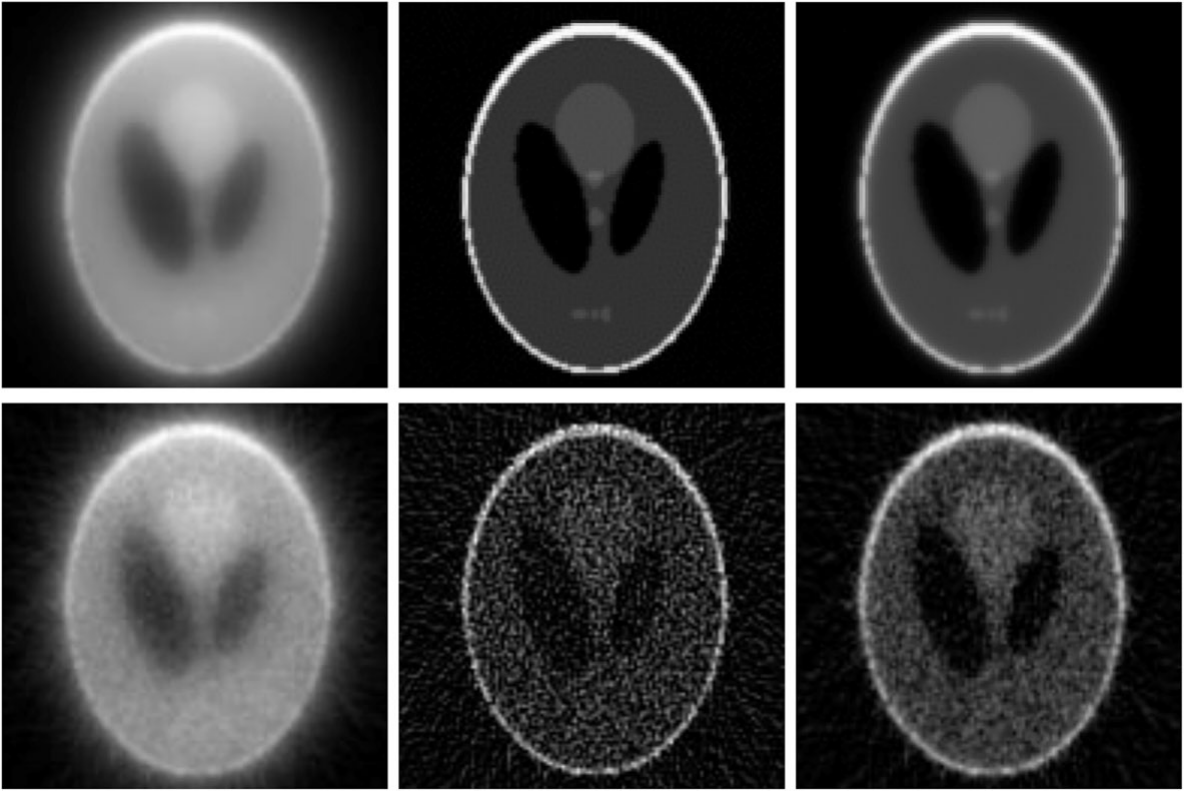
Two-dimensional reconstructions. Upper row: Noiseless simulations; Lower row: Noisy simulations; Left column: Equivalent time-of-flight backprojection; Middle column: Backprojection filtering (BPF) reconstructions; Right column: BPF reconstructions with noise control window function applied

A 2D post filter of *H*(*ω*)*W*(*ω*) was applied to the backprojected image, where *H*(*ω*) and *W*(*ω*) were defined in Eqs. (14) and (16), respectively. Two sets of reconstructions were produced: one using noiseless data and the other using noisy data. The reconstruction results are shown in Fig. 7, where the noiseless simulations are in the upper row and the noisy simulations are in the lower row. The left column shows the equivalent TOF backprojection of the list-mode data. The middle column shows the reconstruction with the proposed BPF algorithm using the post filter *H*(*ω*). The right column shows the reconstruction with the proposed BPF algorithm using the post filter *H*(*ω*)*W*(*ω*).

We remind the readers that the BPF algorithm is different from the FBP algorithm, which is the popular FBP algorithm, while the BPF algorithm is not as popular.

## Conclusions

The list-mode TOF BPF algorithm has many advantages. It is faster than the iterative algorithms. We have derived psf for the pure list-mode TOF backprojection for the 2D case. Once this psf is obtained, a closed-form expression for the reconstruction filter is obtained for the 2D case. Finally, we discuss how the backprojection profile function and the Gaussian function in the tomography filter are determined. We can assume that the TOF timing uncertainty can be modeled as a Gaussian propability distribution with a standard deviation of σ_1_. The parameter σ_1_ is determined by the PET system we are using. The TOF backprojection profile function can also be assumed to be a Gaussian function with a standard deviation of σ_2_. The system psf as defined in Eqs. (9) and (10) is Gaussian with a standard deviation of σ_3_, which must satisfy σ_3_ = σ_1_ + σ_2_. In Eqs. (9)-(15), the parameter σ is σ_3_. The parameter σ_2_ is only used in the TOF backprojector's profile function and can be arbitrarily chosen. It is an interesting special case that σ_2_ = 0, in which the TOF backprojector simply backprojects an event to a single point in the image domain. In this interesting special case, the backprojector is much faster than the conventional non-TOF backprojector and we have σ_3_ = σ_1_. In conventional non-TOF tomography, we have σ_1_ = σ_2_ = σ_3_ = ∞. Computer simulations are performed to test the feasibility of the proposed algorithm. Our immediate future plan is to perform list-mode data simulations, because our simulations in this current paper use non-list-mode data to emulate the list-mode TOF effects by using the central slice theorem. The modern PET can be operated in its 2D mode or 3D mode. The 3D mode has a higher sensitivity by accepting more photons. The 2D mode, on the other hand, has less scattering problem. If the data is acquired using the 3D mode, a 2D data set can be obtained via rebinning, for example, the Fourier rebinning. The reconstruction algorithm for the 3D case is much more complicated than that for the 2D case. The work for the 3D case will be covered in a future paper.

## Data Availability

Not applicable.

## Abbreviations

1D: One-dimensional
2D: Two-dimensional
3D: Three-dimensional
BPF: Backprojection filtering
FBP: Filtered backprojection
LOR: Line-of-response
PET: Positron emission tomography
ps: Pico-seconds
psf: Point spread function
TOF: Time-of-flight

## References

[1] Tomitani T (1981). Image reconstruction and noise evaluation in photon time-of-flight assisted positron emission tomography. IEEE Trans Nucl Sci.

[2] Conti M, Bendriem B, Casey M, Chen M, Kehren F, Michel C (2005). First experimental results of time-of-flight reconstruction on an LSO PET scanner. Phys Med Biol.

[3] Zeng GL (2012). A filtered backprojection algorithm with characteristics of the iterative Landweber algorithm. Med Phys.

[4] Zeng GL (2014). Model based filtered backprojection algorithm: a tutorial. Biomed Eng Lett.

[5] Zeng GL (2010). Medical image reconstruction: a conceptual tutorial.

[6] Defrise M, Casey ME, Michel C, Conti M (2005). Fourier rebinning of time-of-flight PET data. Phys Med Biol.

[7] Li YS, Defrise M, Matej S, Metzler SD (2016). Fourier rebinning and consistency equations for time-of-flight PET planograms. Inverse Probl.

[8] Gradshteyn IS, Ryzhik IM (1994). Table of integrals, series, and products.

[9] Shepp LA, Logan BF (1974). The Fourier reconstruction of a head section. IEEE Trans Nucl Sci.

